# Surface Plasmon Resonance (SPR)-Based Biosensors as Instruments with High Versatility and Sensitivity

**DOI:** 10.3390/s20113010

**Published:** 2020-05-26

**Authors:** Victoria Shpacovitch, Roland Hergenröder

**Affiliations:** Leibniz Institut für Analytische Wissenschaften-ISAS-e.V., Bunsen-Kirchhoff Straße 11, 44139 Dortmund, Germany; roland.hergenroeder@isas.de

## 1. Introduction

Surface plasmon resonance (SPR), as a physical phenomenon, is not restricted only to events occurring in thin planar metal films. A broad spectrum of differently nanostructured surfaces as well as noble metal nanoparticles are frequently employed for fabrication of SPR-based assays. However, conventional commercial SPR-based biosensors and experimental devices are often represented by instruments, which utilize Kretschmann’s [1] scheme of plasmon excitation.This scheme appears to be the most attractive for planar metal films due to its relative simplicity. In this scheme, a prism with a thin metal film deposited on its base is illuminated by p-polarized light. Surface plasmons (SPs) are excited as propagating electron density waves on the metal-dielectric interface in the presence of p-polarized incidence light at a particular angle. Thus, the energy of a laser beam is transformed into electron-polaritons within thin metal film deposited on prism. SPs excited along the metal-dielectric interface lead to a dramatic reduction of the reflected beam intensity and to formation of reflection conditions, which are very sensitive to any changes taking place in the close proximity to metal-dielectric interface. Certainly, among such changes is the adsorption of molecules onto the metal surface and formation the layers of molecules. Thus, SPR-based biosensors can be employed to characterize interactions between biomolecules immobilized onto the metal film sensor surface and their counterparts in liquid sample in real time and without labeling. Indeed, these biosensors are actively used to measure binding constants, kinetics of biomolecular interactions and to perform concentration measurements [2]. In turn, these applications make SPR-based biosensors very useful in pharmacological, biomedical, environmental and food studies.

Nowadays, hundreds research manuscripts describing biological applications of SPR-based biosensors are published annually. However, there are still many challenges in this dynamic research field, which need to be solved. Thus, the presented Special Issue aimed to highlight not only the diversity of biological applications of SPR-based biosensors, but also to indicate novel trends in engineering of SPR-based sensing platforms. Each manuscript of this special issue was a subject of peer-reviewing process.

## 2. Summary of the Special Issue

In the work [3], an SPR imaging (SPRI)-impedance sensor was developed. This sensor enables the detection of refractive index (RI) and impedance changes on a sensor chip. The optical scheme of this sensor resembled Kretschmann’s configuration apart from specific modifications (sensor chip is a glass slide coated with comb-shaped thin gold film). Living basophilic leukemia cells were cultured on the sensor chip and cells were activated with a dinitro-phenol-conjugated human serum albumin (DNP-HSA) antigen. DNP-HSA antigen is often used for sensitization and priming of leukocytes. Application of this immunogen led to cell degranulation and morphological changes [3], which were assessed for cells cultured on the sensor chip. In turn, developed SPRI-impedance sensor helped to monitor simultaneously changes of living-cell derived refractive index (RI) and impedance. In general, RI changes can indicate alterations in the distribution of cellular molecules and ions in the SPR detection area (the depth of SPR evanescent field, which is commonly less than 500 nm). On the other hand, impedance changes are associated with changes in cellular morphology. Thus, analyzed simultaneously, RI and impedance provide more detailed information regarding cellular status than any of them alone.

In the works [4,5], SPR-based assays were employed for pathogen detection and monitoring the traces of fungicide in samples. These works highlighted the potential of SPR-based approaches in agricultural sciences. A home-made SPR-based biosensor [4] was developed for the detection of fungus *P. fijiensis* in samples of banana leaf extracts. The sensor in [4] employed the Kretschmann’s scheme of surface plasmon excitation. This instrument enabled the detection of fungal banana disease at early stages indicating a potential of SPR-based sensor as an alternative analytical tool for monitoring of plant diseases. On the other hand, not only early diagnostic of fungal diseases but also the monitoring of residual fungicides in agricultural products is important for agricultural and food sciences. A SPR-based assay suggested in the work [5] helps to detect residual fungicide thiophanate methyl in samples. In this work, home-made gold nanospheres were employed to enhance Raman signal for the detection of residual thiophanate methyl in samples. LOD of this method was determined as 17 μM of thiophanate methyl.

The work [6] can be considered as a continuation of the previous work published in *Sensors* [7] and describing the employment of SPR-based sensor for sizing and quantification of individual biological nano-particles (virus-like particles, viruses or extracellular vesicles). The previous work [7] introduced the Plasmon Assisted Microscopy of Nano-sized Objects (PAMONO) sensor as a biosensor for quantification and sizing of single microvesicles and virus-like particles (VLPs). PAMONO sensor utilizes Kretschmann’s scheme of plasmon excitation for label-free and specific detection of biological nano-particles. However, if previously in [7] the detection and sizing of nano-particles was performed using Convolutional Neural Networks (CNNs), in the work presented in this special issue [6] frequency domain analysis approach was used for the characterization of nano-paricles. The employment of frequency domain analysis is less computation intensive in comparison with application of CNNs for nano-particle characterization. This feature is especially important for further development of PAMONO sensing platform as a point-of-care diagnostic unit (in hospitals or laboratories with limited resources, which require relatively fast sensing systems). More detailed information about optical and analytical characteristics of the PAMONO sensor can be also found in the series of works [8,9,10,11].

In the works [12,13,14], the improvement of sensitivity of SPR-based sensors was under intensive investigation. In [12], the authors used gold nanowires (AuNW) in order to increase the sensitivity of LSPR-based optical fiber refractive index sensor and optimized its parameters. The work [13] aims to overcome inherent weak points of SPR-sensors, which utilize silver film for plasmon excitation. SPR sensors based on silver films are known to produce relatively sharp intensity curve and this feature can be beneficial for sensor sensitivity and selectivity. However, silver film is highly susceptible to oxidation and this issue can dramatically affect SPR signal. To overcome this limitation, the authors [13] developed planar plasmonic substrate employing an atomic MoS${}_{2}$ layer on a silver surface. Production of a large-area MoS${}_{2}$ monolayer helped to limit oxidation of silver film and, as the authors demonstrated theoretically and experimentally, served to improve the sensitivity of SPR sensor [13]. In the work [14], the authors built up and investigated characteristics, primarily sensitivity, of SPR-based sensors constructed via deposition of multilayer (Nb${}_{2}$O${}_{5}$/SiO${}_{2}$/TiN) structures on the flat surface of D-shaped prism.

Finally, we would like to thank all the authors who contributed their works in order to launch this special issue. We are also deeply grateful to reviewers for their very hard work, which certainly improved the quality of the prepared Special Issue.
